# Bipolar disorder and grey matter heterotopia : a case report

**DOI:** 10.1192/j.eurpsy.2022.1634

**Published:** 2022-09-01

**Authors:** E. Bergaoui, M. Zrelli, N. Staali, M. Moalla, R. Lansari, W. Melki

**Affiliations:** Razi Hospital, Psychiatry D, Manouba, Tunisia

**Keywords:** grey matter heterotopia, bipolar disorder

## Abstract

**Introduction:**

The grey matter heterotopias are a relatively common group of conditions characterized by interruption of normal neuronal migration from near the ventricle to the cortex. Subependymal grey matter heterotopia, also known as periventricular heterotopia, is the most common form.

**Objectives:**

To search a link between bipolar disorder and grey matter heterotopia

**Methods:**

A case report of a woman with grey matter heterotopia who is diagnosed as bipolar

**Results:**

A 34 year old woman was admitted at Razi psychiatric hospital 3 months after childbirth. She was agitated, logorrheic with multiple projects and insomniac. The diagnosis was a manic episod with a marked score of 28/44 at The Bech-Rafaelsen Mania Scale (MAS). The patient was treated with 4 mg of risperidone and 1000 mg of sodium valproate with partial remission after two weeks. One month after her discharge, she had depressive mood, asthenia, anhedonia and insomnia. She had a score of 19 at Hamilton Depression Rating Scale (HDRS). She was switched from risperidone to olanzapine 15mg/j with partial remission after two weeks. In front of persistent symptoms with labile mood, she took lithium 1000 mg/j. She was complaining of a headache and a fluctuating heaviness of the right upper limb. At brain imaging, she had periventricular nodular heterotopia. The patient was adressed to neurology department.

**Conclusions:**

Grey matter heterotopia can cause a variety of neuropsychiatrc symptoms which can lead to diagnosis difficulties. Therefore, in front of atypical symptoms or drug-resistance, patients should be referred for brain imaging.

**Disclosure:**

No significant relationships.

